# Highly conductive anion exchange membranes based on polymer networks containing imidazolium functionalised side chains

**DOI:** 10.1038/s41598-021-83161-9

**Published:** 2021-02-12

**Authors:** Ebrahim Abouzari-Lotf, Mohan V. Jacob, Hossein Ghassemi, Masoumeh Zakeri, Mohamed Mahmoud Nasef, Yadollah Abdolahi, Ali Abbasi, Arshad Ahmad

**Affiliations:** 1 Advanced Materials Research Group, Center of Hydrogen Energy, Institute of Future Energy, Universiti Teknologi Malaysia, 54100 Kuala Lumpur, Malaysia; 2 Helmholtz Institute Ulm (HIU) Electrochemical Energy Storage, Helmholtzstraße 11, 89081 Ulm, Germany; 3 Electronics Materials Lab., College of Science and Engineering, James Cook University, Townsville, QLD 4811 Australia; 4 Department of Macromolecular Science & Engineering, Case Western Reserve University, Cleveland, 44106-7202 USA; 5 Malaysia-Japan International Institute of Technology, Universiti Teknologi Malaysia, Jalan Sultan Yahya Petra, 54100 Kuala Lumpur, Malaysia; 6 Nano Chemical Engineering Department, Shiraz University, Shiraz, Iran; 7 Advanced Materials for Energy Storage, Chulalongkorn University, Bangkok, Thailand

**Keywords:** Chemistry, Energy science and technology, Materials science, Nanoscience and technology

## Abstract

Two novel types of anion exchange membranes (AEMs) having imidazolium-type functionalised nanofibrous substrates were prepared using the facile and potentially scalable method. The membranes’ precursors were prepared by graft copolymerization of vinylbenzyl chloride (VBC) onto syndiotactic polypropylene (*syn*-PP) and polyamide-66 (PA-66) nanofibrous networks followed by crosslinking with 1,8-octanediamine, thermal treatment and subsequent functionalisation of imidazolium groups. The obtained membranes displayed an ion exchange capacity (IEC) close to 1.9 mmol g^–1^ and ionic (OH^-^) conductivity as high as 130 mS cm^–1^ at 80 °C. This was coupled with a reasonable alkaline stability representing more than 70% of their original conductivity under accelerated degradation test in 1 M KOH at 80 °C for 360 h. The effect of ionomer binder on the performance of the membrane electrode assembly (MEA) in AEM fuel cell was evaluated with the optimum membrane. The MEA showed a power density of as high as 440 mW cm^−2^ at a current density is 910 mA cm^−2^ with diamine crosslinked quaternized polysulfone (DAPSF) binder at 80 °C with 90% humidified H_2_ and O_2_ gases. Such performance was 2.3 folds higher than the corresponding MEA performance with quaternary ammonium polysulfone (QAPS) binder at the same operating conditions. Overall, the newly developed membrane was found to possess not only an excellent combination of physico-chemical properties and a reasonable stability but also to have a facile preparation procedure and cheap ingredients making it a promising candidate for application in AEM fuel cell.

## Introduction

The interest in the development of new anion exchange membranes (AEMs) has been recently growing faster than proton exchange membranes (PEMs) counterparts for fuel cell applications^1^. This is due to their advantages promoting lesser fuel permeation, quicker electrochemical reactions, and wider options of cheaper electrocatalysts. Having such merits may lead to a dramatic reduction in the cost per kilowatt of power in such fuel cell devices. Despite the number of technical achievements that have been made pertaining the enhancement of the conductivity and chemical stability of AEMs, the current membrane generations are still far from reaching levels desired for commercialization of AEM fuel cells^2–4^. Particularly, the critical issues demanding further investigations in the quest for new membranes mainly include lack of sufficient alkaline stability of both ionic groups and polymer substrates and the absence of long-range phase-separated ionic nanodomains in the membranes together with the lack of simple preparation procedures that can be used to produce AEMs in larger quantities^3,5–7^.

The manipulation of the polymer morphology towards construction of continuous ion transport pathways in the membrane by polymer modifications has been widely considered to improve the conductivity and enhance the stability of AEMs^8–13^. Such modifications were mainly achieved by crosslinking of the polyelectrolyte^14–16^, formation of hydrophobic/hydrophilic block co-polymers^8,17,18^, pore-filling of porous substrates with cationic electrolyte^19,20^, enhancement of the channel orientation by employing a DC electric field while casting the polymer solution^21^ and the use of nanofibrous substrate to host cationic electrolytes^22^. Particularly, nanofibrous composite films were evaluated for fabrication of highly conductive and robust AEMs following the pioneering work for the preparation of nanofibrous composite PEMs by Pintauro and coworkers^23–25^. The nanofibrous composite membranes demonstrated exclusive microstructural properties including high specific surface area, high volume fraction of void space and flexible porosity, which improve the pore interconnectivity and provide an extensive interface between the two phases present in the membranes^22,26,27^. The combination of the hydrophobic fibrous structure and the side hydrophilic ionic chains is likely to develop phase-separated morphology constructing continuous ion conducting pathways in these membranes boosting the ionic conductivity. The mechanical properties of such nanofibrous structured-membranes can be improved by crosslinking. Alteration of membrane’s morphology was also attained by modification of the ionic side chains of the polymers by introducing various spacers^11^ and multi-cationic side chains^9,11^. Typically, it was observed that the length of alkyl spacers between the cationic groups directly affects the water uptake and conductivity of AEMs^11^. On the other hand, reducing the degree of functionalisation while increasing the ion exchange capacity (IEC) thru incorporation of double-, triple-, quadruple- and poly-cations per side chain were found to balance hydroxide conductivity and alkaline stability while lowering the water uptake of AEMs^9,28–32^. Such changes were ascribed to the formation of well-developed phase separation and larger ionic clusters induced by the incorporation of poly-cationic side chains. Interestingly, the activation energy for the ion transport in AEMs of polycationic side chains was found to be comparable with perfluorinated sulfonic acid (PFSA) membranes under humidified conditions suggesting a promising approach for alternative highly conductive and stable membranes^9^.

Several cationic groups such as quaternary ammonium, pyridinium, benzimidazolium, pyrrolidinium, phosphonium, guanidinium, morpholinium and imidazolium have been considered for functionalisation of AEMs to achieve desired stability^33,34^. Of all, imidazolium cations have been investigated as functional groups in AEMs using different approaches in various occasions^35–40^. Particularly, studies focusing on reduction of the degradation of imidazolium cations having *p*-conjugated imidazole ring by SN2 substitution or Hofmann elimination reactions with hydroxide ions revealed that the presence of substitutions at C2, C4 and C5 positions creating steric effect enhanced the alkaline stability^41–46^. Earlier, radiation grafted AEMs having 2,3-dimethylimidazolium head-groups prepared by a simplified method demonstrated insufficient chemical stability in 1 M KOH at 60 °C^47^. Thus, it is highly interesting to further improve the conductivity and alkaline stability of radiation grafted AEM taking into consideration that such parameters are highly affected not only by well-defined morphology of the membrane and chemical structures but also by the alkaline media^37–40,48^.

In this study, novel AEMs with imidazolium-type functionalised groups grafted onto substrates with nanofibrous morphologies are reported. C2-substituted imidazolium-based chains were introduced to the nanofibrous substrates after their mechanical integrity was improved by crosslinking of the grafted side chains. The properties of the obtained composite membranes such as ion exchange capacity, ionic conductivity, swelling and water uptake, mechanical properties, thermal resistance, and alkaline stability were evaluated. The most conductive and stable membrane was used to fabricate membrane-electrode-assembly (MEA) and tested in an alkaline fuel cell operated at 80 °C with H_2_ and O_2_ feed gases. The morphology of the obtained membranes with the nanofibrous structure and π-conjugated stable imidazole ring having long polycationic side chains is expected to enhance the phase separation in the membranes, improve the stability and subsequently enhance their fuel cell performance.

## Experimental section

### Materials

Vinyl benzyl chloride (VBC) monomer composed of *meta*- and *para*-isomers was purchased from Sigma-Aldrich. *Syndiotactic* polypropylene (*syn*-PP) (M_w_ = 174,000; M_n_ = 75,000), polyoxyethylene sorbitan monolaurate (Tween-20), 1,2-dimethylimidazole (Im), 1,8-octanediamine (ODA) and decalin were all analytical grade and purchased from Sigma-Aldrich. All other reagents were also having analytical grade and used as received. Technical grade polyamide (PA-66, medium viscous) was purchased from DSM Co. Binders of quaternary ammonium polysulfone (QAPS) with IEC of 1.1 mmol g^−1^ and diamine cross-linked quaternized polysulfone (DAPSF) with IEC of 1.60 mmol g^−1^ were prepared according to procedures reported in the literature^49–51^. *Syn*-PP (335 nm mean fibres diameter) and PA-66 (90 nm mean fibres diameter) nanofibrous networks were prepared following the procedure described in the next section and reported elsewhere^9,52,53^. Pt/C (40%, Johnson Matthey Co.) and PtRu/C (50% Pt and 25% Ru, Johnson Matthey Co.) electrocatalysts were used to assemble the membrane electrode assembly (MEA).

## Preparation of membranes

### Nylon and syn-PP nanofiber preparation

Electrospun substrates with different thicknesses were prepared by electrospinning for various time periods (8–40 h) under optimum conditions for PA-66 (18 wt% polymer in formic acid and water mixture (9/1, v/v), applied voltage of 20 kV, tip to collector distance of 12 cm, flow rate of 0.4 mL h^−1^ and collector drum rotation speed of 200 r min^−1^) and *syn*-PP (7.5 wt% polymer in decalin, acetone and dimethylformamide mixture (8/1/1, v/v), applied voltage of 16 kV, tip to collector distance of 20 cm, flow rate of 4 mL h^−1^ and collector drum rotation speed of 500 r min^−1^). The obtained PA-66 and *syn-PP* nanofibrous networks were dried in a vacuum oven for 12 h to completely remove the solvent residues. Nanofibrous networks of PA-66 with the mean diameter of 90 nm and *syn-PP* of mean diameter of 335 nm.

#### Graft copolymerization of VBC onto nanofibrous networks

Graft copolymerization of VBC was carried out by radiation induced emulsion grafting (RIEG) method using preirradiated samples. The nanofibrous substrates of different thicknesses were first irradiated under vacuum in a sealed polyethylene bag by an electron beam accelerator (NHV Nissin high voltage, EPS 3000) to a total dose of 300 and 35 kGy for PA66 and *syn*-PP, respectively. The acceleration voltage was 2 meV and the beam current was 10 mA. A grafting emulsion mixture composed of 5 wt% of VBC monomer and 0.5 wt% of Tween-20 (emulsifier) in distilled water was prepared and bubbled with purified N_2_ to remove air. The deaerated emulsion was transferred into an evacuated glass ampoule containing the irradiated nanofibrous substrate and the ampoule was sealed before it was heated in a water bath at 50 °C for 5 h to perform the graft polymerization. The grafted nanofibrous substrates were extracted and repeatedly washed with deionized water (DI) and methanol followed by drying in a vacuum oven for 10 h at 50 °C. The extent of graft copolymerization or degree of grafting (dg) was evaluated based on the weight increase according to Eq. (1).

1 $$dg \left(\mathrm{\%}\right)=\frac{{\mathrm{W}}_{g}-{\mathrm{W}}_{s}}{{\mathrm{W}}_{s}}\times 100$$

where, W_s_ and W_g_ are the weights of the nanofibrous substrates before and after grafting, respectively.

#### Crosslinking of PVBC grafted substrates

The PVBC grafted nanofibrous substrates were crosslinked with ODA to improve their strength and dimensional stability. The crosslinking was carried out using the procedure reported in a previous study^9^. A 0.3 g of PVBC grafted nanofibrous network with *dg* of 60% was immersed in a 5% solution of ODA, which was heated to 50 °C under stirring for 30 min. To reduce the possibility of ODA attachment from one side, the nanofibrous networks were removed, washed thoroughly with DI water, and transferred into the new flask containing DI water and heated under reflux for 5 h. The crosslinked substrates were removed, washed with DI water a few times, and dried in a vacuum oven at 50 °C for 24 h. The extent of crosslinking (*cd*) was determined based on the following equation after converting into OHˉ form^9^.

2 $$cd \left(\mathrm{\%}\right)=\frac{({\mathrm{IEC}}_{n.c}-{\mathrm{IEC}}_{c})}{{\mathrm{IEC}}_{\mathrm{n}.\mathrm{c}}}\times 100$$

where, the subscript ‘*n.c’* refers to the non-crosslinked (not treated with diamine after grafting) and ‘*c’* refers to the crosslinked substrates. PVBC grafted nanofibrous network with ‘*cd*’ in the range of 2.0–6.4% were obtained.

#### Conversion of nanofibrous sheets into dense membranes

A two-step procedure involving swelling in a solvent vapour followed by a mechanical compression was used to convert the porous nanofibrous substrates into dense membranes. Particularly, the crosslinked PVBC grafted nanofibrous substrate was exposed to tetrahydrofuran (THF) vapour for 1 h and the swollen substrate was mechanically compressed between two ETFE sheets at 87 °C with a pressure of 2 MPa for 10 s. The process was repeated 4 times and the samples were rotated 90° three times to ensure a uniform compression.

#### Introduction of imidazolium group and conversion into OHˉ form

A sample of 0.3 g of crosslinked PVBC grafted substrate was immersed in a 100 mL solution of 2 wt% 1,2-dimethylimidazole in acetonitrile. The temperature was increased to 85 °C and maintained for 24 h. Subsequently, the membrane was removed, washed with DI water and methanol to remove the excess of imidazole followed by drying under vacuum at 50 °C overnight. The membrane was immersed in N_2_ bubbled 1 M KOH aqueous solution for 48 h to replace Clˉ with OH^−^ form prior to MEA fabrication. The residual KOH was removed by washing in DI water several times and the membrane was finally stored in deaerated DI water.

### Evaluation of membranes properties

The morphologies of the composite membranes and their respective precursors were evaluated by Philips XL30 field emission scanning electron microscope (FE-SEM) after sputtering with 5 nm of Au. The cross-sectional morphology was examined using cryo-fractured samples after dipping in liquid nitrogen. The chemical composition with respect to elemental analysis of C, H and N and chemical groups was investigated using LECO CHNS-932 Elemental Analyzer and Thermo Fisher Scientific Nicolet iS50 Fourier transform spectrometer, respectively. BET (Brunauer–Emmett–Teller) analysis was performed in a relative desorption/adsorption pressure range of 0.025–0.997 using an automated high-resolution gas sorption system (Autosorb iQ-MP/XR, Quantachrome Instruments). Thermal properties and thermal behaviour of the samples were investigated by DSC-60 Plus differential scanning calorimeter (DSC) and TGA 55 thermal gravimetric analyser (TGA) using samples dried in a vacuum oven at 70 °C for 24 h. TGA runs were made in a temperature range of 50–500 °C at a constant heating rate of 10 °C min^−1^ under air whereas DSC runs were taken in the range of 30–300 °C at a constant heating rate of 10 °C min^−1^ under nitrogen atmosphere. The mechanical properties of the membranes were measured with respect to tensile strength (MPa) elongation at break (%) using a universal mechanical tester (Shimadzu AGS-X). The membrane samples of 4 cm × 1 cm were equilibrated in liquid water at 25 °C for 2 h and their surface were plotted with tissue paper prior to the tests.

#### Ion exchange capacity

The ion-exchange capacity of the developed membranes was measured in terms of theoretical (IEC_theo_), experimental (IEC_exp_) and accessible (IEC_acc_) values. The IEC_exp_, which is defined in *mmol* of Clˉ per *g* of dried polymer was determined by the Mohr titration method whereas the IEC_theo_ was calculated based on the grafting yield (*dg*) and the assumption of achieving 100% amination. On the other hand, the IEC_acc_ is defined as the portion of accessible IEC fraction and was calculated from the ratio of theoretical and the experimental values of IEC.

#### Ionic conductivity

Through-plane ionic conductivity of the membranes was measured with a conductivity testing device (MTS-740, Scribner Associates Inc.), allowing wet and dry gas mixing for accurate and precise RH control and rapid RH cycling, coupled with an impedance analyser (PSM1735 FRA). An oscillating voltage of 10 mV over a frequency range of 10^6^–10 Hz was applied, and the OH^-^ conductivity was measured in the temperature range of 30–80 °C. The membrane samples were compressed between two gas diffusion layers (E-TEK ELAT GDL 140-HT) and attached to the platinum electrodes with conductive carbon paint with a compression of 2.1 MPa. The samples were equilibrated for 4 h before performing the measurements at desired temperature under continuous purified N_2_ gas flow and constant 90% relative humidity. Z Plot software (Scribner Inc.) was used to fit the impedance plots to an equivalent circuit and the area specific resistance of the membranes (ASR_membrane_) was determined from the high frequency intercept (R_HF_) according to the following equation.

3 $${ASR}_{membrane}={(R}_{HF}\times {A}_{eff})-{ASR}_{cell}$$

where, A_eff_ is the effective sample area (in cm^2^) and ASR_cell_ is the cell resistance, which was determined by recording the uncorrected ASR of membranes with various thicknesses (14, 30, 50 and 60 μm) followed by drawing the values vs membrane thickness data and extrapolation to the theoretical zero membrane thickness by linear regression^54^. Finally, the OH^-^ conductivity of membrane σ_membrane_ was calculated according to Eq. (4):

4 $${\upsigma }_{membrane} (\mathrm{S}.{\mathrm{cm}}^{-1})= \frac{\mathrm{L }\left(\mathrm{cm}\right)}{{\mathrm{ASR}}_{membrane}{\left(\Omega .\mathrm{cm}\right)}^{2}}$$

where, L is the thickness of the membrane.

#### Water uptake and swelling behaviour

Membrane water content and hydration play important role in the performance of anion exchange membranes. The water uptake (ϕ_w_) of the membranes in OH^−^ form, which is the difference in the weights before (W_dry_) and after soaking the membranes in the deaerated water for 24 h (W_wet_), was calculated using the following equation.

5 $${\phi }_{w}=\frac{{W}_{wet}-{W}_{dry}}{{W}_{dry}}\times 100$$

Measurements of the membranes’ dimensions were performed to calculate the swelling ratios in terms of in-plane (${S}_{\parallel}$) and through-plane (${S}_{\bot}$) ratios using Eq. (6).

6 $${\mathrm{S}}_{\mathrm{x}}=\frac{{\mathrm{X}}_{\mathrm{wet}}-{\mathrm{X}}_{\mathrm{dry}}}{{\mathrm{X}}_{\mathrm{dry}}}\times 100$$

where, x represents in-plane or through plane swelling measurements, whereas X_wet_ and X_dry_ are the thicknesses or lengths of swollen and dry membranes, respectively.

Hydration number or water-uptake coefficient of the membranes (λ) was calculated as the number of moles of water per the number of moles of quaternary amine according to the following equation.

7 $$\uplambda =\frac{\mathrm{n}\left({\mathrm{H}}_{2}\mathrm{O}\right)}{\mathrm{n}\left(-{\mathrm{N}}^{+}\right)}=\frac{{\mathrm{W}}_{\mathrm{wet}}-{\mathrm{W}}_{\mathrm{dry}}}{{\mathrm{W}}_{\mathrm{dry}}} \times \frac{1000}{{\mathrm{M}}_{{\mathrm{H}}_{2}\mathrm{O}}\times {\mathrm{IEC}}_{\mathrm{exp}}}$$

where, ${\mathrm{M}}_{{\mathrm{H}}_{2}\mathrm{O}}$ is the molecular mass of water (18.015 g mol^−1^).

#### Chemical stability

The chemical stability was evaluated in the accelerated degradation condition of 1.0 M KOH as the membranes were immersed for up to 360 h at 80 °C and the changes in the ionic conductivity and IEC values were closely monitored. In order to compare with other materials, the stability factor (Κ), which is the ratio of conductivity at 80 °C after 12 days of immersion in strong alkaline condition to the original value at the same temperature was calculated according to Eq. (8).

8 $$K=\frac{{\sigma }_{288 h}}{{\sigma }_{0h}}\times 100$$

#### Fuel cell test

Selected membrane in the hydroxide form was used to fabricate the MEA for single H_2_/O_2_ fuel cell test. QAPS or DAPSF ionomers was used as a binder and the catalyst ink was prepared by ultrasonic treatment of the mixture of catalyst and 9 wt% ionomer in an aqueous solution of isopropyl alcohol. The ink was applied by spraying onto a surface of carbon paper (Toray TGPH-030) using a Badger 150 airbrush (Badger Air-Brush co.) until achieving a catalyst loading level of 0.5 mg cm^−2^ for both anode and cathode sides. MEAs with an active area of 1 cm^2^ was prepared without using hot-pressing. Fuel cell test was conducted with PEMFC test station (850e, Scribner Associates Co.) at 80 ℃ and no back pressure was applied. After preheating of the cell and setting the gas humidifiers under N_2_ flow, the gases fed to the cell were switched to 90% humidified H_2_ and O_2_ with respective flow rates of 100 and 200 mL min^−1^ until reaching a stable OCV prior to taking the reading.

## Results and discussion

### Synthesis, characterization, and membrane fabrication

**Figure 1:**
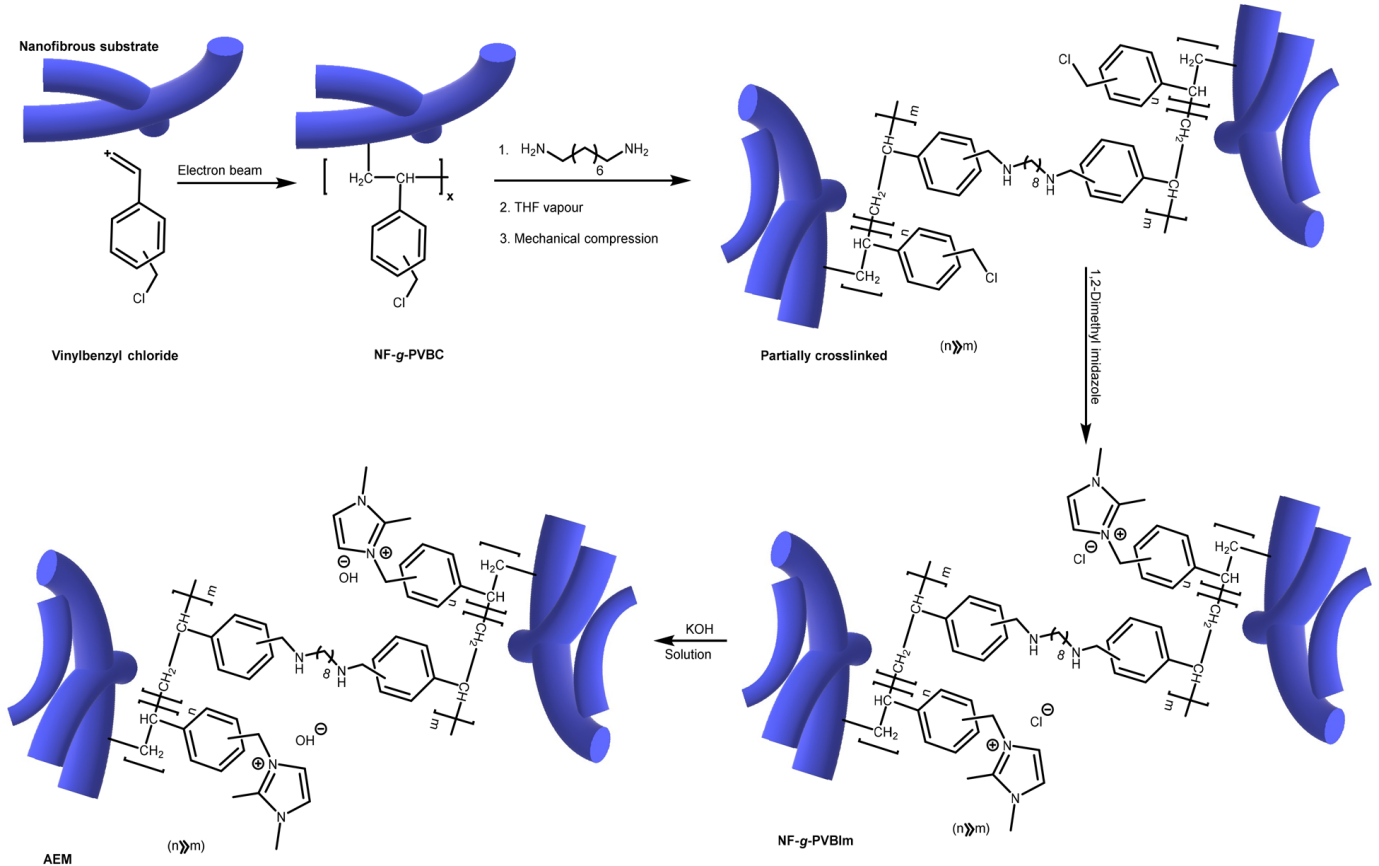
Mechanism for preparation of AEM containing imidazolium functionalised, PVBC grafted and crosslinked nanofibrous membranes.

Figure 1 shows the pathway for preparation of the imidazolium containing AEMs involving grafting, crosslinking and subsequent functionalisation. Based on our previous results^9^; graft copolymerization of PVBC onto PA-66 and *syn*-PP with a *dg* of around 60% could be simply achieved by RIEG. As mentioned earlier, the mechanical integrity of the substrates was enhanced by crosslinking prior to the introduction of imidazoluim groups. Crosslinking step was manipulated in a way that allowed controlling the *cd* by variation of the amount of ODA in the reaction. In the subsequent treatment step, 1,2-dimethylimidazole acted as a nucleophile and attacked benzyl group to replace chloride atom. The reaction variables were optimized to achieve quantitative substitution. The crosslinked PA-66-*g*-PVBIm and *syn*-PP-*g*-PVBIm were converted to dense membranes by swilling in THF vapor and compressing. The chloride ions in the obtained dense membranes were exchanged for hydroxide by immersion in KOH aqueous solution.

**Figure 2:**
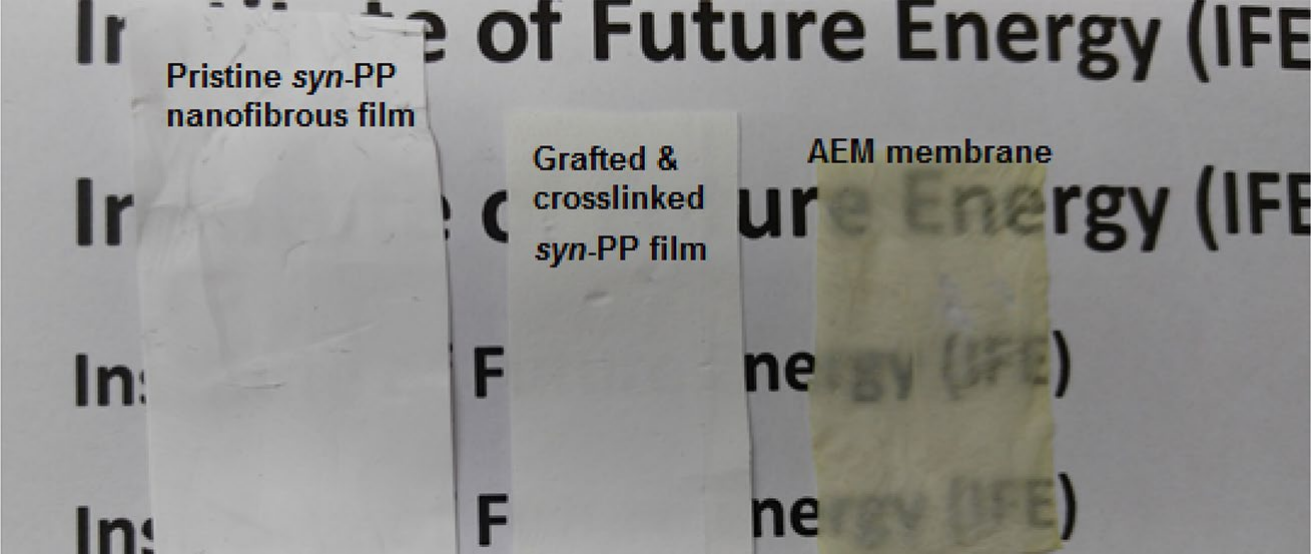
Images of pristine *syn*-PP, crosslinked *syn*-PP*-g-*PVBC *and syn*-PP-*g*-PVBCIm (AEM) membrane.

Figure 2 shows images of pristine *syn*-PP, crosslinked *syn*-PP*-g-*PVBC *and syn*-PP-*g*-PVBCIm (AEM) membrane. It can be obviously seen the white colour of the pristine and the grafted nanofibrous substrates became transparent after functionalisation of the membrane with imidazolium groups.

**Figure 3:**
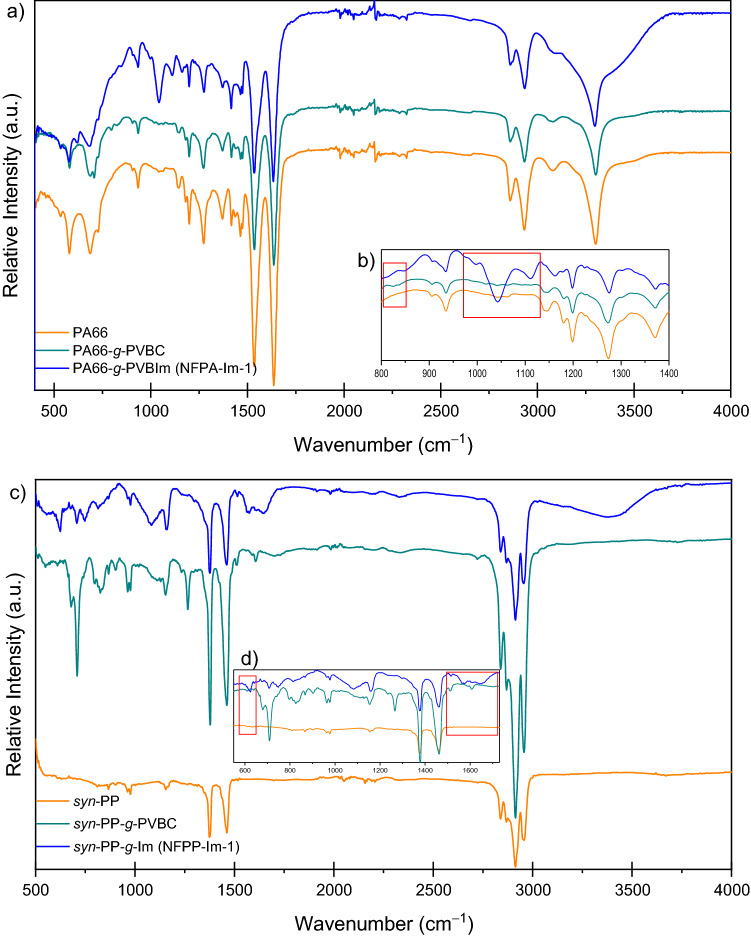
FTIR-ATR spectra of pristine nanofibers, NF-*g*-PVBC and NF-*g*-PVBIm (hydroxide form) of PA-66 (**a**) and *syn*-PP (**c**) based substrates and corresponding expanded regions (**b**) and (**d**).

Figure 3 shows FT-IR spectra of pristine nanofibers, nanofiber-*g*-PVBC and nanofiber-*g*-PVBIm membranes in OH^-^ form. Compared to pristine *syn*-PP, PVBC grafted samples showed aromatic C-H peaks of *para*-substituted ring of VBC in the region of 790–840 cm^−1^. On the other hand, meta-linked di-substitution showed few bands in the regions of 650–700 and 860–900 cm^−1^. However, in the case of PA-66 grafted substrates such peaks were overlapped by the polyamide-related peaks. In *syn*-PP based substrates, a specific band for methylene wagging mode of phenyl-CH_2_-Cl was observed at 1265 cm^−1^ confirming the grafting of PVBC on the nanofibrous substrates. The presence of benzyl and –Cl ions are necessary to observe such a wagging mode band as reported in the literature^55^. The complete replacement of –Cl atom by di-substituted Im was confirmed by the complete disappearance of the band at around 1265 cm^−1^. For the nanofiber-*g*-PVBIm samples, the IR spectrum clearly shows the characteristic peaks for out-of-plane and in-plane-bending of C-H of the imidazole ring in the region of 1050–1100 cm^−1^. Moreover, N–H and O–H stretching vibration were appeared as a broad band at around 3400 cm^−1^ in the Im substituted substrates.

In addition to IR analysis, elemental analysis confirmed the successful grafting and crosslinking of the PVBC side chains as per the data summarized in Table 1. Typically, PA-66 showed the elemental percentages of 62.95 for C, 9.94 for H and 12.36 for N. Upon grafting, the elemental contributions were changed to 67.04, 7.99 and 6.50% for C, H and N, respectively. Such changes are going along with the theoretical calculations for 60% grafted substrates with elemental chemical formula of (C_12_H_22_N_2_O_2_)_0.53n_(C_9_H_9_Cl)_0.47n_.

**Table 1:** Elemental composition and results of BET analysis of imidazolium functionalised membranes together with pristine nanofibers and PVBC grafted substrates.

Sample	Formula		Elemental composition (%)^a^			BET analysis results		
			C	H	N	Specific surface area (m^2^ g^−1^)	Average pore diameter (nm)	Pore volume (cm^3^ g^−1^)
PA-66	(C_12_H_22_N_2_O_2_)_n_	C	63.68	9.80	12.38	36.64 ± 3.2	140 ± 15.2	0.086 ± 0.0075
		F	62.95	9.94	12.36			
*syn*-PP	(C_3_H_6_)_n_	C	85.63	14.37	–	2.00 ± 0.16	45 ± 4.1	0.022 ± 0.0023
		F	85.52	14.42	0.01			
PA-66-*g*-PVBC	(C_12_H_22_N_2_O_2_)_0.53n_ (C_9_H_9_Cl)_0.47n_	C	67.04	7.99	6.50	8.25 ± 0.85	38 ± 4.0	0.017 ± 0.0020
		F	66.33	8.04	6.50			
*syn*-PP-*g*-PVBC	(C_3_H_6_)_0.86n_ (C_9_H_9_Cl)_0.14n_	C	83.56	13.19	–	1.42 ± 0.15	25 ± 2.2	0.010 ± 0.0011
		F	83.41	13.22	0.02			
PA-66-*g*-PVBIm (NFPA-Im-1)	–	F	68.14	7.12	7.32	2.01 ± 0.18	22 ± 1.9	0.011 ± 0.0010
*syn*-PP-g-PVBIm (NFPP-Im-1)	–	F	84.26	14.73	0.12	1.00 ± 0.09	16 ± 1.8	0.006 ± 0.0005

^a^“C” refers to calculated values and “F” refers to found values based on elemental analysis.

The morphology and pore structure of the two types of substrates were substantially changed upon grafting, crosslinking and functionalisation as prevailed in the data shown in Table 1. For example, specific surface area, average pore diameter and pore volume of PA-66 nanofibrous substrate were reduced from original values of 36.64 (m^2^ g^−1^), 140 (nm) and 0.086 (cm^3^ g^−1^) to 8.25 (m^2^ g^−1^), 38 (nm) and 0.017 (cm^3^ g^−1^), respectively. The crosslinking of the grafted PA-66 affected the porosity to great extent and yielded a reduction in the specific surface area, average pore diameter and pore volume by approximately 75, 42 and 35%, respectively. Similar decrease in pore structure properties were observed in the membrane after functionalisation.

**Figure 4:**
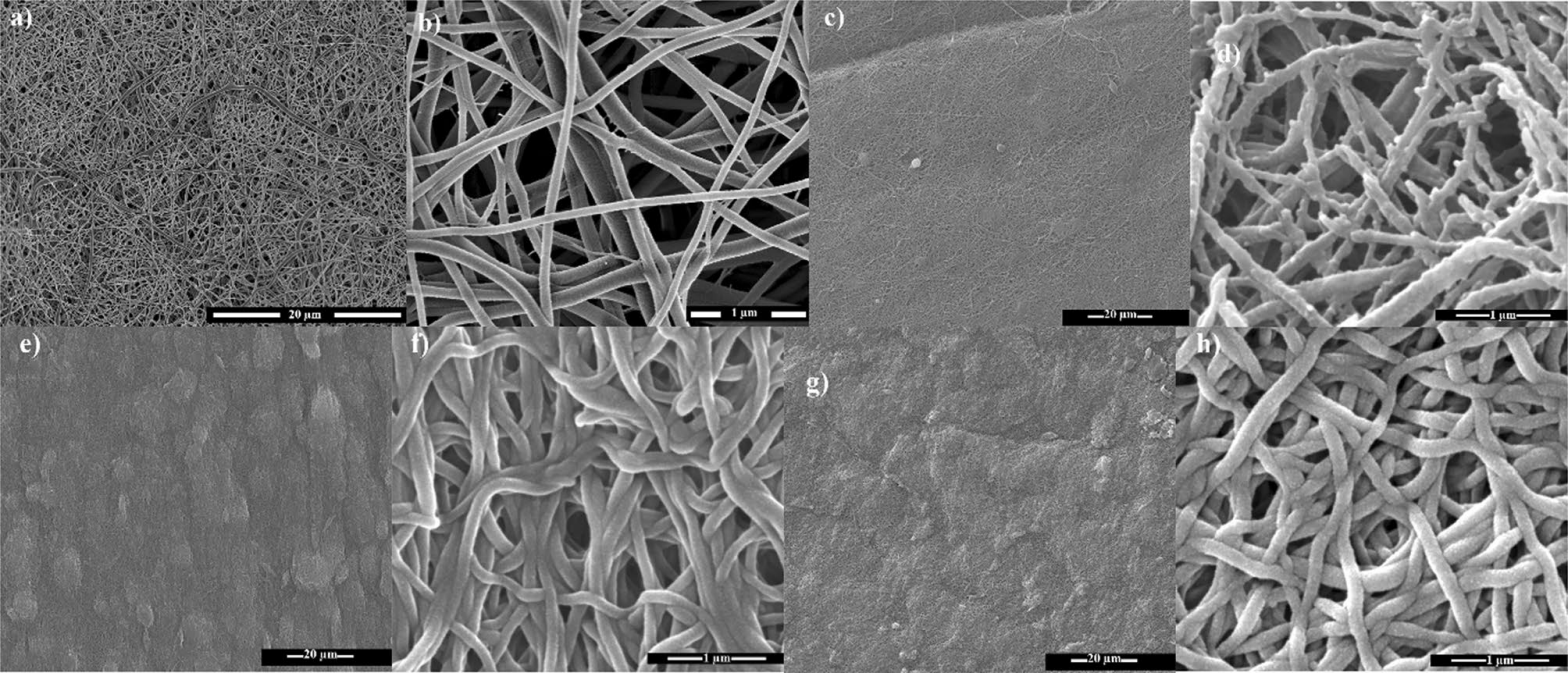
FE-SEM images of pristine PA-66 nanofiber (**a**, **b**), PVBC grafted substrates (**c**, **d**), PVBIm of Clˉ (**e**, **f**) and PVBIm of OHˉ forms (**g**, **h**) in various magnifications.

The SEM images of pristine PA66 nanofibers (a, b), graft copolymerized substrates (c, d), PVIm functionalised in the Clˉ (e, f) and OHˉ forms (g, h) are shown in Fig. 4. As can be clearly seen, the morphology of the nanofibrous substrates grow denser upon grafting, crosslinking, and introduction of Im group as depicted in the low- and high-resolution SEM images.

**Figure 5:**
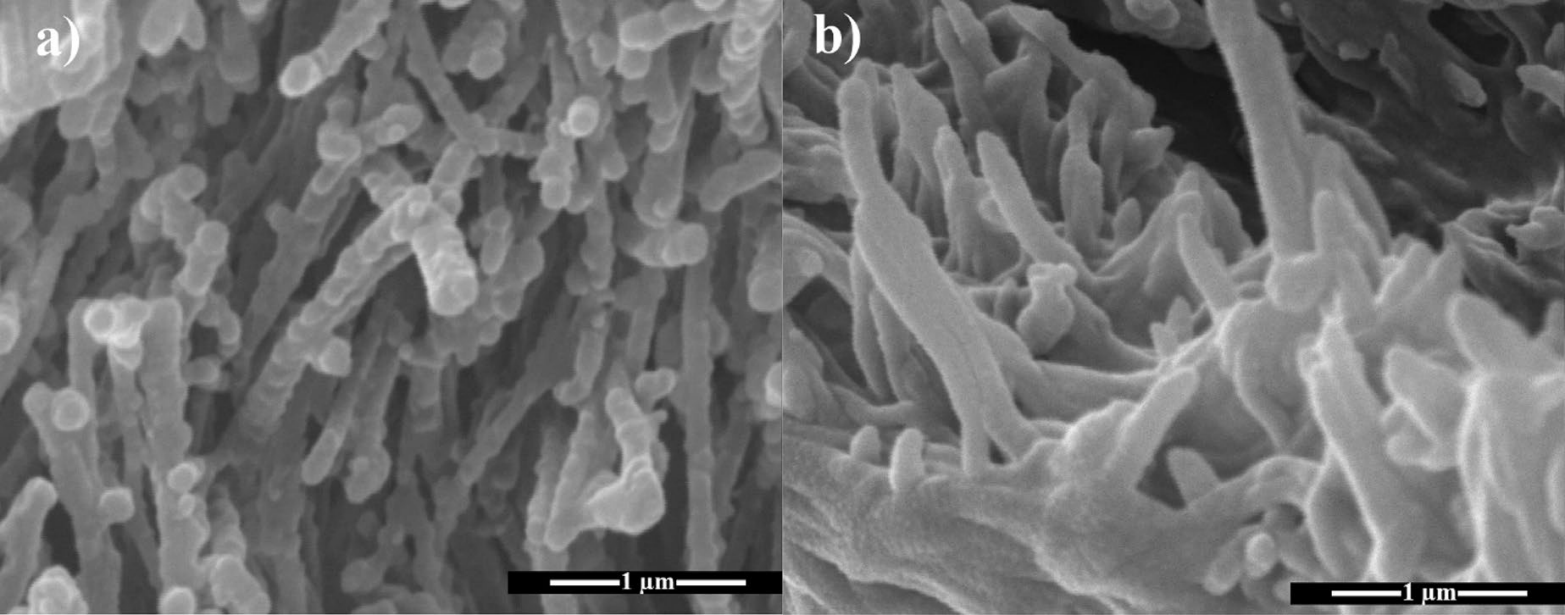
Cross-sectional views of PA66-*g*-PVBIm substrates before (**a**) and after (**b**) swelling by THF vapor exposure.

The efficiency of the exposure to THF vapor to convert nanofibre-*g*-PVBIm substrate (in the Clˉ form) to the dense membrane after compression could be similarly seen in the cross-sectional view of SEM images presented in Fig. 5.

**Figure 6:**
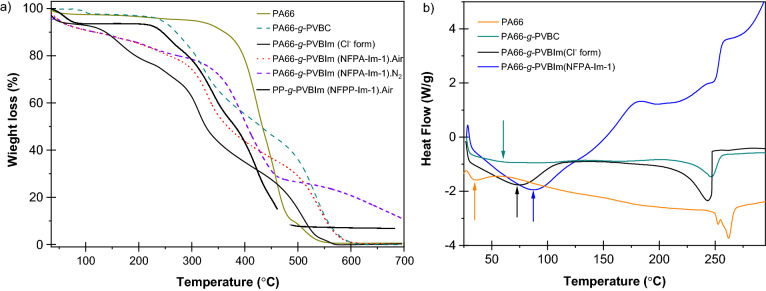
TGA thermograms (**a**) and DSC thermograms (**b**) of PA66, PA66-*g*-PVBC and PA66-*g*-PVBIm in the Clˉ and OHˉ forms. The thermal stability of PP based membrane (PP-g-PVBIm-1) is added in (**a**) for comparison.

The thermal behaviour and stability of the membranes in the Clˉ and OHˉ forms together with their intermediate substrates were investigated and typical TGA and DSC thermograms for PA-66 based substrates are presented in Fig. 6. The degradation patterns of original nanofibrous networks (shown in Fig. 6a), PVBC and PVBIm grafted substrates involved one-, two- and three-transition steps, respectively. The nanofibre-*g*-PVBIm showed 7–10 wt% weight loss at 150 ℃ despite prior drying under vacuum at 60 °C and this due to the prolonged loss of water bound to the samples by H-bonding, which was more prominent in the membranes with OHˉ form and is significantly higher in PA-66 based membranes compared to PP-based membranes. The heat flows associated with melting and glass transition are also evaluated and a typical example of PA-66 based substrates is shown in the DSC thermograms illustrated in Fig. 6b. As can be observed, the melting temperature of PA-66 at 252 °C was reduced to 244 and 246 °C upon grafting with PVBC and introduction of ImCl, respectively. Interestingly, the membrane in OHˉ form showed a broad melting range around 200 °C. An opposite behaviour was observed for the glass transition temperature where the T_g_ of the pristine PA-66 was increased from 35 to 60 °C upon grafting and from to 72 and 87 °C for the membrane in the forms of Clˉ and OHˉ forms, respectively.

### Water uptake and ion exchange capacity of membranes

Both water uptake and swelling degree are closely related to the exchangeable ions in the polymeric membranes, which directly affect the ionic conductivity and the overall fuel cell performance. Table 2 shows the values of IEC, water uptake, λ values and swelling ratios of the developed AEMs of various thicknesses and crosslinking densities compared to their analogues membranes with trimethylammonium (TMA) groups. The IEC_theo_ calculated taking into considering the *dg* (60%) and assuming 100% amination were 2.02 and 2.09 mmol g^–1^ for PA-66 based membranes having *cd* of 5.6 and 2.3%, respectively. Similarly, for *syn*-PP based membranes with *a dg* of 60% and *cd* of 6.5 and 3.3%, the IEC_theo_ values were 2.00 and 2.07 mmol g^–1^, respectively. Interestingly, the IEC_exp_ values were at very close range with theoretical counterparts and the IEC_acc_ portion was as high as 95% for all membranes, which indicates that almost all anion exchange groups are accessible. Such extraordinary accessibility could possibly be due to the phase-separated morphology developed in the present membranes. Table 1 also showed that the membranes have relatively lower water uptake, λ, and swelling ratios especially with higher *cd*. This is caused by the restricted segmental motion accompanied by the formation of polymer network structure because of crosslinking.

**Table 2:** Ion exchange capacity and swelling ratios of developed membranes.

Sample	Thickness (μm)^*a*^	*cd*^b^ (%)	IEC (mmol g^–1^)		IEC_acc_^c^ (%)	ϕ_w_ (%)	Λ	Swelling ratios (%)^d^	
			IEC_theo_	IEC_exp_				${S}_{\mathrm{\parallel}}$	${S}_{\bot}$
NFPA-Im-1	14 ± 0.3	5.6	2.09	2.04 ± 0.03	97	50.5 ± 0.5	13.7	26	29
NFPA-Im-2	22 ± 0.5	5.6	2.09	2.05 ± 0.03	98	50.0 ± 0.6	13.5	26	30
NFPA-Im-3	14 ± 0.3	2.3	2.02	1.92 ± 0.02	95	59.5 ± 0.5	17.2	26	33
NFPA-Im-4	22 ± 0.4	2.3	2.02	1.92 ± 0.02	95	60.1 ± 0.5	17.4	29	32
NFPA-TMA-1	15 ± 0.3	5.6	2.02	1.86 ± 0.03	92	72.4 ± 0.8	21.6	33	36
NFPA-TMA-2	17 ± 0.3	2.3	2.09	1.94 ± 0.02	93	85.3 ± 0.8	24.4	36	37
NFPP-Im-1	16 ± 0.3	6.5	2.00	1.94 ± 0.03	97	47.3 ± 0.4	13.5	25	26
NFPP-Im-2	17 ± 0.4	3.3	2.07	2.02 ± 0.03	98	52.9 ± 0.4	14.5	28	28
NFPP-TMA-1	15 ± 0.3	6.5	2.00	1.94 ± 0.02	97	70.1 ± 0.6	20.1	38	33
NFPP-TMA-2	17 ± 0.4	3.3	2.07	2.03 ± 0.03	98	81.7 ± 0.8	22.3	31	35

^a^Measured at 10 random points for hydrated membranes using a digital micrometer.

^b^Crosslinking degree.

^c^Accessible ion exchange capacity.

^d^Measured at 30 °C in water.

Moreover, membranes with imidazolium ion exchange groups exhibited lower water uptakes and the number of water molecules per cationic group compared to membranes of similar IECs with TMA groups. However, the reported *λ* range of 13.5–17.4 is competitive with the typical hydrated AEMs found in the literature. It is noteworthy mentioning that while water uptake is required for getting high conductivity in the AEMs, excess water uptake exerts a dilution on the conductive species leading to a reduction in the conductivity in addition to possible delamination from MEA upon swelling change. This also might be contributed to the higher IEC_acc_ in *syn*-PP based membranes and those having high *cd*. In addition, higher water uptake directly affects the swelling. For instance, the developed membranes with imidazoluim functionality showed a reduced swelling ratio compared to TMA containing analogous membranes. Such lower swelling ratios are beneficial for improving the mechanical stability of the membranes as summarized in Table 3.

**Table 3:** Mechanical properties, ionic conductivities, and their corresponding activation energy of the developed membranes at 30 and 80 °C.

Sample	Average mechanical properties^a^		Conductivity [σ] (mS cm^−1^)		Activation energy kJ mol^−1^
	Tensile strength (MPa)	Elongation at break (%)	30 °C	80 °C	
NFPA-Im-1	73 ± 10	36 ± 6	34.7	109.5	20.6
NFPA-Im-2	75 ± 10	38 ± 7	35.5	112.6	20.8
NFPA-Im-3	77 ± 12	39 ± 8	39.4	130.1	20.2
NFPA-Im-4	75 ± 12	38 ± 5	41.3	125.4	19.9
NFPA-TMA-1	73 ± 11	35 ± 7	46.6	126.5	15.9
NFPA-TMA-2	75 ± 10	30 ± 6	48.3	116.1	18.0
NFPP-Im-1	16 ± 1	55 ± 8	45.2	138.2	21.3
NFPP-Im-2	17 ± 2	59 ± 7	48.4	148.6	20.7
NFPP-TMA-1	16 ± 3	58 ± 7	41.3	132.6	21.2
NFPP-TMA-2	–	–	44.6	127.5	18.9

^a^At 25 °C 80% relative humidity.

### Ionic conductivity

**Figure 7:**
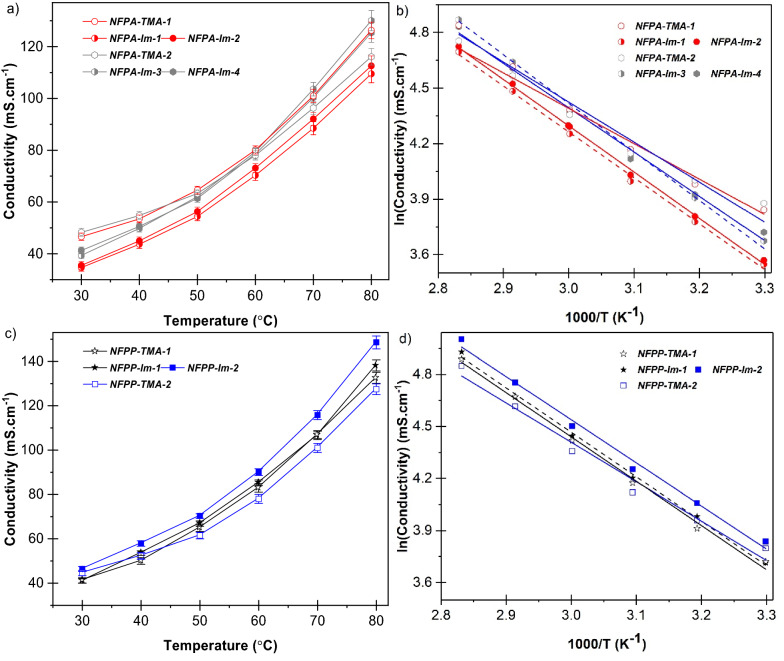
Hydroxide ion conductivity (**a**, **c**) and Arrhenius plot (**b**, **d**) of developed PA-66 (**a**, **b**) and *syn*-PP (**c**, **d**) based membranes as function of temperature compared with TMA functionalised analogous membranes.

The developed membranes in fully hydrated forms exhibited conductivities in the range of 34–149 mS cm^−1^ at a temperature range of 30–80 °C, which are competitive with the typical conductivity values for AEMs reported in the literature^56^. Figure 7 shows the conductivity data of the membranes as a function of temperature (a, c) and the corresponding Arrhenius plot (b, d). The activation energy (*E*_*a*_) values were calculated from the slope of the best linearly fitted data of ln conductivity vs 1/T plot and the values are summarized in Table 3. The conductivity showed a perfect linear behaviour with fitting coefficient (*R*^2^) range of 0.9956–0.9992 and *E*_*a*_ range of 19.9–20.8 kJ mol^−1^. Such low E_a_ values are equivalent to the corresponding values reported for PFSA membranes under fully hydration condition^52^, indicating that there is a facile hydroxide transport in the present nanofibrous-based membranes. When compared to the analogous membranes with TMA groups, the average E_a_ values for membranes with imidazolium functionality are obviously higher and this is likely due to higher water uptake in the former compared to latter membranes. This suggests that the increase in operating temperature will greatly increase OH^-^ transport in membranes with imidazolium group compared to the corresponding ones with TMA. This is evident from conductivity remarkable rise above 60 °C depicted in Fig. 7a and c.

### Stability and fuel cell performance of membranes

Mechanical and alkaline stability of AEMs are critical for their performance and durability in fuel cell. The data for tensile strength and elongation at break for the membranes are presented in Table 3. The average tensile strength was in the range of 73–77 MPa which is higher than that for TMA- containing membranes especially those based PA-66. Such higher values may be attributed to the increase in the intermolecular interactions between PA-66 chains due to the lower water uptake and swelling compared to TMA-containing analogous membranes. Furthermore, the average elongation at break (%) was found to be higher than TMA containing membranes due to the decline in the hydrophilic nature of the imidazolium-containing membranes.

**Figure 8:**
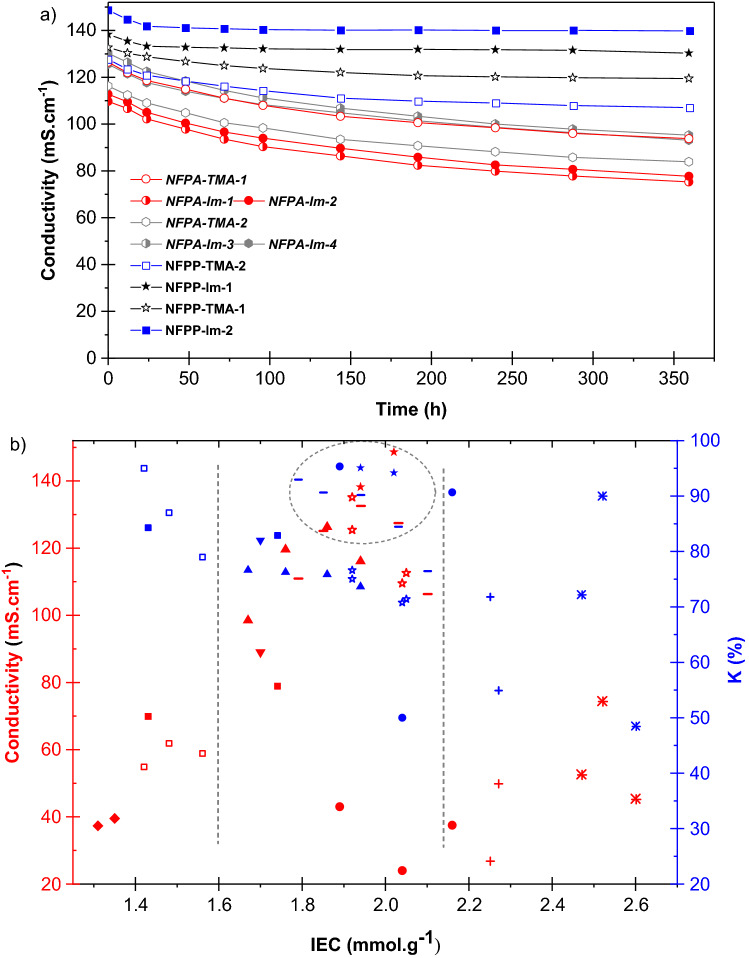
Accelerated alkaline stability of developed membranes at 80 °C (**a**) and comparison of stability factor and conductivity of PA-66 and *syn*-PP-*g*-PVBIm (open star and filled star respectively) membranes with some recently published data (**b**) of PA-66 and *syn*-PP-*g*-PVBTMA (filled triangle and −, respectively)^9^, PVB-TMA (filled diamond)^57^, PPO-TMA with long side-chain (filled inverted triangle, K factor value was reported in 2 M solution)^58^, semi-interpenetrating network (SIPN AEMs) (filled square)^6^, quaternized poly(2,6-dimethyl phenylene oxide) (QAPPO) (filled circle, the conductivity was measured at 20 ℃)^59^, PPO based membrane with one, two and tri-QA group per chain (asterisk, the conductivity was measured at 20 °C)^28^, and crosslinked co-PP (open triangle)^60^.

The chemical stability of the developed membranes was evaluated under an accelerated degradation environment by monitoring the changes in the density of functional groups and ionic conductivity upon immersion in 1 M KOH aqueous solution at 80 °C. The developed imidazolium-containing membranes lost up to 16% of their original IEC values after 15 days of continuous exposure to the alkaline medium while the TMA-containing analogous membranes showed lower stability under the same condition as indicated by 20% loss in their IECs. Figure 8a shows the ionic conductivity decrease over time during the chemical stability test and Fig. 8b compares the conductivity and stability factor with the corresponding values of recently reported AEMs. As can be noticed, all membranes display loss of conductivity over time, which appears to be faster at the beginning of the test. For example, the initial conductivity of 125.4 mS cm^–1^ for NFPA-Im-4 membrane at 80 °C dropped to 104.6 mS cm^–1^ after 144 h and continued to gradually decline to 92.8 mS cm^–1^ until the end of experiment after 360 h.

**Figure 9:**
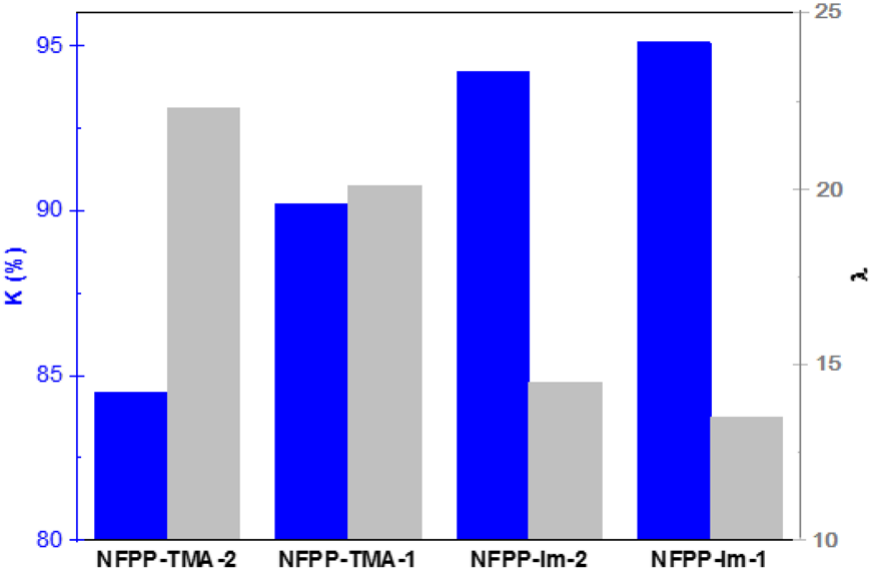
The trend of stability dependence to λ value in the *syn*-PP based substrates.

NFPA-Im membranes were shown to be the least stable material and retained around 70% of their original conductivity after 360 h. Obviously, one can observe two main patterns in Fig. 8a. Firstly, the main difference in the stability is arising from the difference of the host polymer. For instance, the amide groups in PA-66 are more prone to hydrolysis under highly alkaline environment and elevated temperature compared to *syn*-PP counterpart. Secondly, the chemical composition of the ionic side chains results in obvious differences in stability. Interestingly, *syn*-PP and PA66 based substrates showed different degradation behaviour in relation to the ionic side chains. While imidazolium groups seem to be less stable in the case of PA-66 based substrates, an opposite behaviour was noticed in the *syn*-PP based substrates since the membranes with imidazolium were more stable than membranes with TMA functionality. This is in contrast with other reports that showed higher inherent stability for quaternary ammonium groups when compared with the 1,2-dimethlimidazolium ionic groups^43,45,61^. In fact, the stability factor in *syn*-PP based membranes is more dependent on the λ than on the ionic groups as shown in Fig. 9. One possible explanation for this trend is that the extent of exposure of ionic groups to the hydroxide ions is also playing a role affecting the stability of the membranes in a way directly boosting the chance for degradation at the expense of the inherent stability of the ion exchange groups. This fact was mainly neglected in the previous studies pertaining AEM stability and degradation.

**Figure 10:**
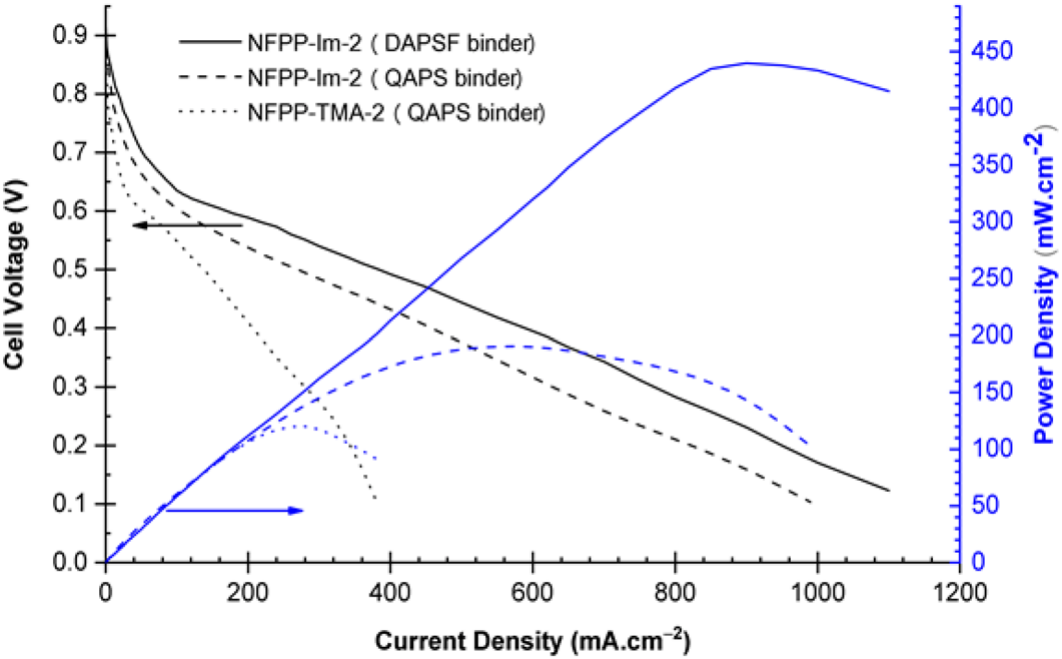
Polarization curve and power density of a single fuel cell with MEAs prepared using of NFPP-Im-2 membranes with different binders in comparison with NFPP-TMA-2 membrane. Conditions are: T = 80 °C, RH = 90% for H_2_ and O_2_ having flow rates of 100 and 200 mL min^−1^, respectively.

The performance of selected samples of NFPP-Im-2 membrane having a thickness of 17 µm was tested in MEA assembled using either DAPSF or QAPS ionomer binder. MEA with a membrane based on the same substrate but containing tetramethylammonium groups (TMA) from a previous study^9^ assembled using QAPS binder and denoted as NFPP-TMA-2 was used for comparison. The cell was operated at 80 °C using 90% humidified H_2_ and O_2_ and the obtained polarization curve and power density are presented in Fig. 10. The open circuit voltage (OCV) of the prepared MEAs with DAPSF and QAPS under the present cell operating conditions was found to be 0.90 and 0.87 V, respectively. These values are just below the desired values (1.1 V) for this type of fuel cells and indicate the need for improving the gas barrier property in the developed membranes by increase the thickness. The membrane in MEA with QAPS binder showed a peak power density of 190.7 mW cm^−2^ at a current density of 570 mA. Interestingly, the peak power density increased by 2.3 folds reaching 440 mW cm^−2^ at a current density is 910 mA cm^−2^ for the MEA with DAPSF binder. These observations suggest that the presence of high ionic conductivity was encountered by a lower resistance to ionic transport in the electrode with DAPSF compared to that of QAPS ionomer binder. This is going along with the ion conductivity trend, which is higher in the former ionomer than the latter. On the other hand, MEA with NFPP-TMA-2 membrane and QAPS binder showed far inferior power density (110 mW cm^−2^ at a current density of 280 mA cm^−2^) than that in the present study. This indicates that the membrane prepared in this study has superior performance compared to that in Ref.^9^ even though both membranes have similar thicknesses and IEC (as shown in Tables 2 and 3). It can be concluded that the incorporation of ionomer binder with higher conductivity in MEA enhanced the anion transport in the catalyst layer of the electrode, and reduced the interfacial resistance leading to a substantial improvement in the performance of AEM fuel cell.

## Conclusion

Two types of AEMs were successfully prepared by RIEG of VBC onto nanofibrous networks of PA66 and *syn*-PP followed by compression and subsequent functionalisation with imidazoluim groups. Crosslinking was used to enhance the dimensional stability and mechanical properties of the membranes. The new membranes with thicknesses in the range of 14–20 μm and IEC range of 1.92–2.04 mmol g^–1^ exhibited superior ionic conductivity and mechanical strength compared to the corresponding membranes with TMA ionic groups regardless the type of nanofibrous substrate. A significant correlation between the hydration number and stability factor was established in *syn*-PP based membranes. Of all membranes, NFPP-Im-2 was found to have the best combination of physico-chemical properties and thus it was chosen for further evaluation in AEM fuel cell. A promising fuel cell performance was achieved in a single cell test at 80 °C where MEA with NFPP-Im-2 membrane showed high power density of 190.7 mW cm^−2^ and OCV of around 0.87 V with QAPS ionomer binder which is 1.7 times higher than that with NFPP-TMA-2 membrane counterpart (110 mW cm^−2^ at a current density of 280 mA cm^−2^). The incorporation of DAPSF ionomer binder having higher conductivity in the MEA increased OCV to 0.90 V and the peak power density by 2.3 folds reaching a value of 440 mW cm^−2^ at a current density is 910 mA cm^−2^. This improvement is caused by the enhancement in the OH^-^ diffusion in the catalyst layer of the electrode and the reduction of the ohmic loss associated with the interfacial resistance between the membrane and electrode. However, the OCV needs to be further improved by enhancing the gas barrier property through increasing the thickness of the membranes. Overall, the performance of the imidazolium-functionalised nanofibrous membranes that was coupled with an excellent combination of physico-chemical properties and reasonable stability provides promising candidates for AEM fuel cell with thicker thickness. Moreover, the simplicity of the preparation procedure and inexpensive chemicals used for making these membranes confer them a strong upscaling potential.
